# The combined effect of pre-pregnancy body mass index and gestational weight gain on the risk of pre-labour and intrapartum caesarean section—The ICE-MCH study

**DOI:** 10.1371/journal.pone.0280060

**Published:** 2023-01-05

**Authors:** Aino-Maija Eloranta, Ingibjörg Gunnarsdottir, Birna Thorisdottir, Geir Gunnlaugsson, Bryndis Eva Birgisdottir, Inga Thorsdottir, Kristjana Einarsdóttir

**Affiliations:** 1 Institute of Public Health and Clinical Nutrition, School of Medicine, University of Eastern Finland, Kuopio, Finland; 2 Institute of Biomedicine, School of Medicine, University of Eastern Finland, Kuopio, Finland; 3 Department of Medicine, Endocrinology and Clinical Nutrition, Kuopio University Hospital, Kuopio, Finland; 4 Faculty of Food Science and Nutrition, School of Health Sciences, University of Iceland, Reykjavík, Iceland; 5 Unit for Nutrition Research, Health Science Institute, University of Iceland and Landspitali University Hospital, Reykjavík, Iceland; 6 Unit for Nutrition Research, Health Science Institute, University of Iceland, Reykjavik, Iceland; 7 Faculty of Sociology, Anthropology and Folkloristics, School of Social Sciences, University of Iceland, Reykjavík, Iceland; 8 Faculty of Medicine, School of Health Sciences, University of Iceland, Reykjavík, Iceland; 9 Centre of Public Health Sciences, Health Science Institute, University of Iceland, Reykjavík, Iceland; Aga Khan University pakistan, PAKISTAN

## Abstract

Women who are obese before pregnancy have a higher risk of caesarean section than normal weight women. We investigated the combined effect of pre-pregnancy weight and gestational weight gain on pre-labour and intrapartum caesarean section risk. We collected data on 22,763 singleton, term, live deliveries in 2003–2014 from the Icelandic Maternal and Child Health Study (ICE-MCH), based on Icelandic registries. These were the Icelandic Medical Birth Registry and the Saga Maternal and Child Health Database. Pre-pregnancy body mass index was categorised into underweight, normal weight, overweight and obese. Gestational weight gain was classified according to the Institute of Medicine´s recommendation into below, within and above the recommended range. Logistic regression models, adjusted for maternal and gestational characteristics, were used to calculate adjusted odds ratios (AOR) and 95% confidence intervals (CI) for the risk of caesarean section. Obese women had a higher risk of pre-labour (AOR 1.56, 95% CI 1.34–1.81) and intrapartum caesarean section (AOR 1.92, 95% CI 1.70–2.17) than normal weight women in all categories of gestational weight gain. Gestational weight gain above the recommended range, compared to within the range, increased the risk of intrapartum caesarean section among normal weight (AOR 1.46, 95% CI 1.23–1.73) and overweight women (AOR 1.291, 95% CI 1.04–1.60). Gestational weight gain below the recommended range, compared to within the range, increased the risk of pre-labour caesarean section (AOR 1.64, 95% CI 1.20–2.25), but only among overweight women. Women who are obese before pregnancy have a high risk of caesarean section regardless of gestational weight gain. However, women who are normal weight or overweight before pregnancy and gain weight above the recommended range during pregnancy may also have an increased risk of caesarean section.

## Introduction

The caesarean section rate has increased in most middle- and high-income countries in recent decades [1]. Caesarean section is known to increase the risk of delivery complications for the mother [2] and the newborn [3]. It can also increase the risk of complications in subsequent pregnancies [4] and the risk of health concerns, such as obesity, in offspring from childhood to adulthood [5, 6]. Moreover, the caesarean section appears to be more costly than vaginal delivery in most cases [7]. Therefore, it is essential to identify risk factors for cesarean sections, particularly modifiable ones, to minimise the rate of cesarean sections.

Previous studies show that a higher pre-pregnancy body mass index (BMI) is associated with a range of pregnancy and delivery complications [8] and that overweight and obese women are at an increased risk of caesarean section [9, 10]. The Nordic recommendation for gestational weight gain is lower for overweight and obese women than for normal weight women, set by the Institute of Medicine [11] and adopted by the Nordic Nutrition Recommendations [12]. Gestational weight gain above these recommended ranges [11, 12] has been associated with a higher risk of caesarean section [13, 14]. Some studies have compared the independent effects of pre-pregnancy BMI and gestational weight gain and found that overweight and obesity seem more strongly associated with the risk of caesarean section than excess gestational weight gain [15, 16]. However, studies investigating the effect of gestational weight gain across all categories of pre-pregnancy BMI on the risk of caesarean section are few and have not come to a consensus on the interplay of effects [17–20]. Moreover, it is not well known whether the impact of pre-pregnancy BMI and gestational weight gain differ on pre-labour and intrapartum caesarean sections.

Therefore, it is essential to fully explore the combined effect of pre-pregnancy BMI and gestational weight gain to understand how to counsel women to reduce the rate of caesarean sections. The aim of this study was to investigate the combined effect of pre-pregnancy BMI and gestational weight gain on the risk of pre-labour and intrapartum caesarean section in Iceland.

## Methods

### Data sources and study population

We obtained data of singleton term deliveries (≥37 weeks of pregnancy) for the years 2003–2014 from the Icelandic Maternal and Child Health Study (ICE-MCH 2002–2015), which is based on Icelandic health registries. The registries used in the current study were the Icelandic Medical Birth Registry, which includes information on all deliveries in Iceland, and the Saga Maternal and Child Health Database, which provides information on maternal health visits at Primary Health Care centres during pregnancy.

From the Icelandic Medical Birth Registry, we obtained data on mode of delivery, the onset of labour, multiple gestation, gestational age, maternal age at delivery, parental cohabiting, maternal employment status, parity, and maternal diagnoses. Mode of delivery and maternal diagnoses were registered according to the International Classification of Diseases, 10^th^ revision (ICD-10). Caesarean sections (ICD-10 codes O82.0 and O82.1) were categorised according to the timing of the procedure, i.e., whether it was performed before or after commencement of labour (pre-labour or intrapartum, respectively). For maternal diagnoses, information on pre-pregnancy essential hypertension (ICD-10 code O10.0), pre-pregnancy type 1 or type 2 diabetes (ICD-10 codes O24.0 and O24.1), and pre-eclampsia (ICD-10 codes O14.0, O14.1, O14.2 and O14.9) were also obtained.

We obtained data on the area of residence and maternal body weight and height registered at maternal health visits from the Saga Maternal and Child Health Database. The first visit occurred between gestational weeks 8 and 12, and the measurements of body weight and height at the first visit were used to estimate pre-pregnancy BMI. Pre-pregnancy BMI was calculated as weight divided by height squared (kg/m^2^) and categorised as underweight (<18.5), normal weight (18.5–24.9), overweight (25–29.9) and obese (≥30) [21]. Gestational weight gain was calculated as the difference in body weight between the first and the last maternal health visit and classified according to the Institute of Medicine’s recommended gestational weight gain ranges (Table 1), as above, within or below the recommended weight for their BMI [11].

**Table 1:** Pre-pregnancy Body Mass Index (BMI) and recommended gestational weight gain, as defined by the Institute of Medicine [11].

Pre-pregnancy BMI, kg/m^2^	Pre-pregnancy BMI category	Recommended gestational weight gain, kg
<18.5	Underweight	12.5–18.0
18.5–24.9	Normal weight	11.5–16.0
25–29.9	Overweight	7.0–11.5
≥30	Obese	5.0–9.0

The ICE-MCH study was approved by the National Bioethics Committee in Iceland (VSN-14-078) with later adjustments and the Icelandic Data Protection Authority (ref. 2014050799) and performed in accordance with the Declaration of Helsinki. The Directorate of Health permitted access to information in the Icelandic Medical Birth Registry (ref. 1405034/5.6.1), and the Primary Health Care of the Capital Area (ref. lA3g/22/845.l) and other health care centres in the country permitted access to information in the Saga Maternal and Child Health Database.

### Statistical analyses

We performed all data analyses using the IBM SPSS Statistics software, Version 27.0 (IBM Corp., Armonk, NY, USA). The level of significance was set at P<0.05.

We compared the characteristics across the categories of pre-pregnancy BMI and gestational weight gain using Pearson’s χ^2^ test. We also compared the mean gestational weight gain in the categories of pre-pregnancy BMI using the analysis of variances. Using logistic regression models, we calculated odds ratios (OR) and 95% confidence intervals (CI) for the likelihood of all caesarean sections, pre-labour caesarean sections and intrapartum caesarean sections. The association between pre-pregnancy BMI and caesarean section were modelled with normal weight women as the reference group and stratified by categories of gestational weight gain. Similarly, the association between gestational weight gain and caesarean section were modelled with ‘within recommended weight gain’ as the reference group and stratified by categories of pre-pregnancy BMI. Unadjusted models and models adjusted for maternal age (continuous), pre-pregnancy essential hypertension (yes, no), pre-pregnancy diabetes (yes, no), pre-eclampsia (yes, no), residential area (capital area, outside capital area), parental cohabiting (yes, no), maternal employment status (employed, student, other), parity (primiparous, multiparous), and gestational age (continuous) are presented.

## Results

The study sample consisted of 22,763 singletons, term deliveries in Iceland from 2003 to 2014, of which 13.6% were by caesarean section. The rate of intrapartum caesarean sections was 8.6%, and the rate of pre-labour caesarean sections was 5.0%.

Out of the study sample, 28.9% of women were overweight and 18.5% were obese at gestational weeks 8–12 (Table 2), and 37.0% had gestational weight gain above the recommended range (Table 3). Gestational weight gain was most likely to be above recommended range in overweight women and within the recommended range in normal weight women. Mean (standard deviation) gestational weight gain was 13.2 (5.2) kg in underweight women, 13.2 (5.0) kg in normal weight women, 12.1 (5.5) kg in overweight women and 9.5 (5.6) kg in obese women (P for the difference between groups <0.001).

**Table 2:** Characteristics for all participants and according to pre-pregnancy body mass index categories.

	All	Pre-pregnancy body mass index				
		Underweight	Normal weight	Overweight	Obese	P value^a^
**N (%)**	22,763					
**Pre-pregnancy body mass index**						
Underweight	452 (2.0)					
Normal weight	11,540 (50.7)					
Overweight	6,571 (28.9)					
Obese	4,200 (18.5)					
**Gestational weight gain**						<0.001
Below rec.	6,389 (28.1)	218 (48.2)	4,103 (35.6)	1,155 (17.6)	913 (21.7)	
Within rec.	7,944 (34.9)	165 (36.5)	4,585 (39.7)	1,911 (29.1)	1,283 (30.5)	
Above rec.	8,430 (37.0)	69 (15.3)	2,852 (24.7)	3,505 (53.3)	2,004 (47.7)	
**Gestational age**						<0.001
37–38 weeks	3,236 (14.2)	94 (20.8)	1,659 (14.4)	852 (13.0)	631 (15.0)	
39–40 weeks	13,477 (59.2)	277 (61.3)	6,942 (60.2)	3,891 (59.2)	2,367 (56.4)	
≥41 weeks	6,050 (26.6)	81 (17.9)	2,939 (25.5)	1,828 (27.8)	1,202 (28.6)	
**Maternal age**						<0.001
<20 years	601 (2.6)	37 (8.2)	329 (2.9)	154 (2.3)	81 (1.9)	
20–29 years	11,896 (52.3)	303 (67.0)	6,266 (54.3)	3,258 (49.6)	2,069 (49.3)	
30–39 years	9,654 (42.4)	104 (23.0)	4,672 (40.5)	2,948 (44.9)	1,930 (46.0)	
≥40 years	612 (2.7)	8 (1.8)	273 (2.4)	211 (3.2)	120 (2.9)	
**Residential area**						<0.001
Capital area	15,803 (69.4)	366 (81.0)	8,631 (74.8)	4,334 (66.0)	2,472 (58.9)	
Outside capital area	6,960 (30.6)	86 (19.0)	2,909 (25.2)	2,237 (34.0)	1,728 (41.1)	
**Parental cohabiting**						<0.001
Cohabiting	19,149 (84.1)	345 (76.3)	9,621 (83.4)	5,592 (85.1)	3,591 (85.5)	
Not cohabiting	3,017 (13.3)	90 (19.9)	1,604 (13.9)	829 (12.5)	503 (12.0)	
Unknown	597 (2.6)	17 (3.8)	315 (2.7)	159 (2.4)	106 (2.5)	
**Maternal employment**						<0.001
Employed	18,134 (79.7)	315 (69.7)	9,0891 (78.8)	5,360 (81.6)	3,370 (80.2)	
Student	3,700 (16.3)	98 (21.7)	2,009 (17.4)	982 (14.9)	611 (14.5)	
Other	929 (4.1)	39 (8.6)	442 (3.8)	229 (3.5)	219 (5.2)	
**Parity**						<0.001
Primiparous	9,478 (41.6)	275 (60.8)	5,272 (45.7)	2,461 (37.5)	1,470 (35.0)	
Multiparous	13,285 (58.4)	177 (39.2)	6,268 (54.3)	4,110 (62.5)	2,730 (65.0)	
**Pre-pregnancy essential hypertension**						<0.001
Yes	230 (1.0)	2 (0.4)	48 (0.4)	57 (0.9)	123 (2.9)	
No	22,533 (99.0)	450 (99.6)	11,492 (99.6)	6,514 (99.1)	4,077 (97.1)	
Pre-existing diabetes						<0.001
Yes	24 (0.1)	0 (0.0)	4 (0.0)	3 (0.0)	17 (0.4)	
No	22,763 (99.9)	452 (100.0)	11,536 (100.0)	6,568 (100.0)	4,183 (99.6)	
Pre-eclampsia						<0.001
Yes	655 (2.9)	18 (4.0)	233 (2.0)	204 (2.7)	200 (4.8)	
No	22,108 (97.1)	434 (96.0)	11,307 (98.0)	6,367 (96.9)	4,000 (95.2)	

^a^Difference across categories using Pearson’s χ^2^ test

**Table 3:** Characteristics according to pre-pregnancy gestational weight gain categories.

	Gestational weight gain^a^			
	Below recommended	Within recommended	Above recommended	P value^b^
**N (%)**				
**Pre-pregnancy body mass index**				<0.001
Underweight	218 (3.4)	165 (2.1)	69 (0.8)	
Normal weight	4,103 (64.2)	4,585 (57.7)	2,852 (33.8)	
Overweight	1,155 (18.1)	1,911 (24.1)	3,505 (41.6)	
Obese	913 (14.3)	1,283 (16.2)	2,004 (23.8)	
**Gestational age**				<0.001
37–38 weeks	1,172 (18.3)	1,097 (13.8)	967 (11.5)	
39–40 weeks	3,849 (60.2)	4,763 (60.0)	4,865 (57.7)	
≥41 weeks	1,368 (21.4)	2,084 (26.2)	2,598 (30.8)	
**Maternal age**				<0.001
<20 years	147 (2.3)	174 (2.2)	280 (3.3)	
20–29 years	3,172 (49.6)	3,998 (50.3)	4,726 (56.1)	
30–39 years	2,856 (44.7)	3,525 (44.4)	3,273 (38.8)	
≥40 years	214 (3.3)	247 (3.1)	151 (1.8)	
**Residential area**				<0.001
Capital area	4,588 (71.8)	5,555 (69.9)	5,660 (67.1)	
Outside capital area	1,801 (28.2)	2,389 (30.1)	2,770 (32.9)	
**Parental cohabiting**				0.009
Cohabiting	5,439 (85.1)	6,710 (84.5)	7,000 (83.0)	
Not cohabiting	801 (12.5)	1,025 (12.9)	1,191 (14.1)	
Unknown	149 (2.3)	209 (2.6)	239 (2.8)	
**Maternal employment**				0.365
Employed	5,095 (79.7)	6,360 (80.1)	6,679 (79.2)	
Student	1,015 (15.9)	1,278 (16.1)	1,407 (16.7)	
Other	279 (4.4)	306 (3.9)	344 (4.1)	
**Parity**				<0.001
Primiparous	2,340 (36.6)	3,129 (39.4)	4,009 (47.6)	
Multiparous	4,049 (63.4)	4,815 (60.6)	4,421 (52.4)	
**Pre-pregnancy essential hypertension**				0.984
Yes	65 (1.0)	79 (1.0)	86 (1.0)	
No	6,324 (99.0)	7,865 (99.0)	8,344 (99.0)	
Pre-existing diabetes				0.101
Yes	10 (0.2)	10 (0.1)	4 (0.0)	
No	6,379 (99.8)	7,934 (99.9)	8,426 (100.0)	
Pre-eclampsia				<0.001
Yes	117 (1.8)	177 (2.2)	361 (4.3)	
No	6,272 (98.2)	7,767 (97.8)	8,069 (95.7)	

^a^ Gestational weight gain defined according to the Institute of Medicine [11]

^b^Difference across categories using Pearson’s χ^2^ test

Overweight and obese women were more likely to be older and to live outside the capital area than underweight and normal weight women (Table 2). Obese women were more likely to have been diagnosed with hypertension and diabetes before pregnancy. Obese and underweight women were more likely to be diagnosed with pre-eclampsia in the current pregnancy than normal weight and overweight women. Underweight women were more likely to deliver at lower gestational age, not to cohabit with the other parent, and to be students or not employed than higher-weight women. Women who had gestational weight gain above the recommended range were more likely to be younger and primiparous, deliver at higher gestational age, live outside the capital area, not cohabit with the other parent, and be diagnosed with pre-eclampsia in the current pregnancy than other women (Table 3).

Overweight and obese women had a higher risk of any caesarean section, pre-labour caesarean section, and intrapartum caesarean section than normal weight women (Table 4). Women who had gestational weight gain above the recommended range had a higher risk of any caesarean section and intrapartum caesarean section than women who had gestational weight gain within the recommended range (Table 4). Women who had gestational weight gain below the recommended range had a higher risk of pre-labour caesarean section than women who had gestational weight gain within the recommended range but the association was not evident after adjustment. Instead, women who had gestational weight gain above the recommended range had a higher risk of pre-labour caesarean section than women who had gestational weight gain within the recommended range after adjustment.

**Table 4:** Risk of caesarean section according to pre-pregnancy body mass index and gestational weight gain in 22,763 singleton term deliveries in Iceland during 2003–2014.

	Any caesarean section		Pre-labour caesarean section		Intrapartum caesarean section	
	OR (95% CI)	AOR (95% CI)^a^	OR (95% CI)	AOR (95% CI)^a^	OR (95% CI)	AOR (95% CI)^a^
**Pre-pregnancy body mass index**						
Underweight	0.78 (0.56–1.08)	0.78(0.56–1.09)	0.80 (0.47–1.35)	0.85 (0.50–1.50)	0.79 (0.53–1.17)	0.74 (0.49–1.11)
Normal weight	1.00 (ref.)	1.00 (ref.)	1.00 (ref.)	1.00 (ref.)	1.00 (ref.)	1.00 (ref.)
Overweight	**1.27 (1.16–1.39)**	**1.27 (1.16–1.40)**	**1.27 (1.10–1.47)**	**1.17 (1.01–1.36)**	**1.23 (1.10–1.38)**	**1.29 (1.15–1.45)**
Obese	**1.93 (1.76–2.13)**	**1.91 (1.73–2.11)**	**1.88 (1.62–2.17)**	**1.56 (1.34–1.81)**	**1.82 (1.62–2.04)**	**1.92 (1.70–2.17)**
**Gestational weight gain**						
Below rec.	1.02 (0.93–1.13)	0.99 (0.89–1.09)	**1.18 (1.02–1.37)**	1.01 (0.87–1.18)	0.92 (0.81–1.05)	0.96 (0.84–1.09)
Within rec.	1.00 (ref.)	1.00 (ref.)	1.00 (ref.)	1.00 (ref.)	1.00 (ref.)	1.00 (ref.)
Above rec.	**1.29 (1.18–1.41)**	**1.35 (1.23–1.48)**	1.02 (0.88–1.18)	**1.25 (1.07–1.45)**	**1.44 (1.29–1.60)**	**1.36 (1.21–1.51)**

OR = odds ratio; CI = confidence interval; AOR = adjusted odds ratio; ref. = reference; rec. = recommended gestational weight gain according to the Institute of Medicine [11]

Statistically significant values are bolded.

^a^Adjusted for maternal age, pre-pregnancy essential hypertension, pre-pregnancy diabetes, pre-eclampsia, residential area, parental cohabiting, maternal employment, parity, and gestational age.

Obese women had an increased risk of any caesarean section, i.e., pre-labour caesarean section and intrapartum caesarean section, in all gestational weight categories compared to normal weight women (Table 5). Compared to normal weight women, overweight women had an increased risk of any caesarean section, pre-labour caesarean section, and intrapartum caesarean section only among women below the recommended weight gain range (Table 5). Overweight women also had an increased risk of pre-labour caesarean section among women above the recommended weight gain range, but this association was no longer significant after adjustments.

**Table 5:** Risk of caesarean section according to pre-pregnancy body mass index stratified by gestational weight gain in 22,763 singleton term deliveries in Iceland during 2003–2014.

	Gestational weight gain, according to the Institute of Medicine’s recommendations					
	Below recommendation		Within recommendation		Above recommendation	
	OR (95% CI)	AOR (95% CI)^a^	OR (95% CI)	AOR (95% CI)^a^	OR (95% CI)	AOR (95% CI)^a^
**Any caesarean section**						
Pre-pregnancy BMI						
Underweight	1.08 (0.70–1.66)	1.10 (0.70–1.72)	0.70 (0.39–1.24)	0.72 (0.41–1.29)	0.40 (0.14–1.09)	0.40 (0.15–1.12)
Normal weight	1.00 (ref.)	1.00 (ref.)	1.00 (ref.)	1.00 (ref.)	1.00 (ref.)	1.00 (ref.)
Overweight	**1.67 (1.39–2.01)**	**1.73 (1.43–2.09)**	1.09 (0.92–1.29)	1.05 (0.88–1.25)	1.08 (0.93–1.24)	1.06 (0.92–1.23)
Obese	**2.06 (1.70–2.50)**	**1.99 (1.62–2.44)**	**1.94 (1.64–2.29)**	**1.85 (1.55–2.20)**	**1.66 (1.42–1.93)**	**1.64 (1.40–1.92)**
**Pre-labour caesarean section**						
Pre-pregnancy BMI						
Underweight	1.03 (0.54–1.98)	1.08 (0.55–2.12)	0.57 (0.21–1.55)	0.67(0.24–1.90)	0.41 (0.06–2.94)	0.46 (0.06–3.51)
Normal weight	1.00 (ref.)	1.00 (ref.)	1.00 (ref.)	1.00 (ref.)	1.00 (ref.)	1.00 (ref.)
Overweight	**1.75 (1.34–2.27)**	**1.73 (1.32–2.27)**	1.14 (0.89–1.48)	0.87 (0.67–1.14)	**1.33 (1.03–1.72)**	1.03 (0.79–1.34)
Obese	**1.95 (1.47–2.57)**	**1.71 (1.27–2.30)**	**1.75 (1.35–2.26)**	**1.38 (1.05–1.82)**	**2.16 (1.67–2.81)**	**1.57 (1.19–2.07)**
**Intrapartum caesarean section**						
Pre-pregnancy BMI						
Underweight	1.11 (0.63–1.93)	1.10 (0.63–1.95)	0.80 (0.41–1.58)	0.74 (0.37–1.47)	0.41 (0.13–1.32)	0.38 (0.12–1.23)
Normal weight	1.00 (ref.)	1.00 (ref.)	1.00 (ref.)	1.00 (ref.)	1.00 (ref.)	1.00 (ref.)
Overweight	**1.51 (1.18–1.92)**	**1.59 (1.24–2.04)**	1.05 (0.85–1.29)	1.15 (0.93–1.43)	0.98 (0.83–1.15)	1.07 (0.90–1.27)
Obese	**1.96 (1.53–2.50)**	**1.96 (1.51–2.54)**	**1.91 (1.56–2.34)**	**2.03 (1.63–2.51)**	**1.38 (1.15–1.65)**	**1.57 (1.30–1.89)**

OR = odds ratio; CI = confidence interval; AOR = adjusted odds ratio; ref. = reference; rec. = recommended gestational weight gain according to the Institute of Medicine [11]

Statistically significant values are bolded.

^a^Adjusted for maternal age, pre-pregnancy essential hypertension, pre-pregnancy diabetes, pre-eclampsia, residential area, parental cohabiting, maternal employment, parity, and gestational age.

Women below the recommended range of gestational weight gain had an increased risk of any caesarean section and pre-labour caesarean section if they were overweight at the beginning of pregnancy (Table 6). Women above the recommended range of gestational weight gain were at increased risk of any caesarean section and intrapartum caesarean section if they were normal weight or overweight at the beginning of pregnancy (Table 6).

**Table 6:** Risk of caesarean section according to gestational weight gain stratified by pre-pregnancy body mass index in 22,763 singleton term deliveries in Iceland during 2003–2014.

	Pre-pregnancy body mass index							
	Underweight		Normal weight		Overweight		Obese	
	OR (95% CI)	AOR (95% CI)^a^	OR (95% CI)	AOR (95% CI)^a^	OR (95% CI)	AOR (95% CI)^a^	OR (95% CI)	AOR (95% CI)^a^
**Any caesarean section**								
Gestational weight gain								
Below rec.	1.45 (0.71–2.94)	1.34 (0.64–2.81)	0.94 (0.82–1.08)	0.88 (0.77–1.02)	**1.44 (1.17–1.77)**	**1.44 (1.16–1.78)**	1.00 (0.81–1.24)	0.99 (0.79–1.23)
Within rec.	1.00 (ref.)	1.00 (ref.)	1.00 (ref.)	1.00 (ref.)	1.00 (ref.)	1.00 (ref.)	1.00 (ref.)	1.00 (ref.)
Above rec.	0.72 (0.23–2.29)	0.59 (0.18–1.98)	**1.27 (1.10–1.47)**	**1.35 (1.17–1.57)**	**1.26 (1.06–1.49)**	**1.31 (1.10–1.56)**	1.09 (0.91–1.30)	1.15 (0.96–1.39)
**Pre-labour caesarean section**								
Gestational weight gain								
Below rec.	1.94 (0.60–6.28)	1.50 (0.41–5.47)	1.07 (0.87–1.31)	0.86 (0.69–1.06)	**1.63 (1.20–2.21)**	**1.64 (1.20–2.25)**	1.19 (0.87–1.63)	1.01 (0.72–1.41)
Within rec.	1.00 (ref.)	1.00 (ref.)	1.00 (ref.)	1.00 (ref.)	1.00 (ref.)	1.00 (ref.)	1.00 (ref.)	1.00 (ref.)
Above rec.	0.59 (0.07–5.39)	0.41 (0.04–4.35)	0.83 (0.65–1.06)	1.07 (0.83–1.37)	0.97 (0.75–1.26)	1.30 (0.98–1.71)	1.03 (0.78–1.35)	1.31 (0.99–1.75)
**Intrapartum caesarean section**								
Gestational weight gain								
Below rec.	1.19 (0.50–2.82)	1.17 (0.47–2.90)	0.86 (0.73–1.03)	0.89 (0.75–1.07)	1.24 (0.95–1.63)	1.26 (0.96–1.65)	0.89 (0.68–1.16)	0.94 (0.71–1.24)
`Within rec.	1.00 (ref.)	1.00 (ref.)	1.00 (ref.)	1.00 (ref.)	1.00 (ref.)	1.00 (ref.)	1.00 (ref.)	1.00 (ref.)
Above rec.	0.79 (0.21–3.00)	0.53 (0.12–2.26)	**1.54 (1.30–1.82)**	**1.46 (1.23–1.73)**	**1.43 (1.16–1.76)**	**1.29 (1.04–1.60)**	1.11 (0.90–1.37)	1.04 (0.84–1.16)

OR = odds ratio; CI = confidence interval; AOR = adjusted odds ratio; ref. = reference; rec. = recommended gestational weight gain according to the Institute of Medicine [11]

Statistically significant values are bolded.

^a^Adjusted for maternal age, pre-pregnancy essential hypertension, pre-pregnancy diabetes, pre-eclampsia, residential area, parental cohabiting, maternal employment, parity, and gestational age.

## Discussion

In a population sample of women having singleton, term, live births in Iceland, we found that obese women, compared to normal weight women, had an increased risk of both pre-labour and intrapartum caesarean section regardless of gestational weight gain. Women above the recommended range of gestational weight gain were at increased risk of intrapartum caesarean section if they were normal weight or overweight at the beginning of pregnancy. On the other hand, women below the recommended range of gestational weight gain had an increased risk of pre-labour caesarean section if they were overweight at the beginning of pregnancy, even when maternal and gestational factors were taken into account.

Our finding that obese women had a higher risk of caesarean section compared to normal weight women is in line with previous studies [9, 10]. We found that obese women had an approximately two-fold risk of caesarean section compared to normal weight women. This risk is similar in magnitude to previous meta-analyses [8, 10]. One of the most common causes of pre-labour caesarean section is a previous caesarean section [22]. However, we found that obesity was associated with an increased risk of pre-labour caesarean section also when adjusted for parity. Other possible explanations for the association of obesity with a higher risk of pre-labour caesarean section are decreased success rate in the external cephalic version in the case of breech presentation [23] and fetal macrosomia [24]. Moreover, fetal macrosomia, with excess soft tissue in the mother’s pelvic area causing obstruction, can result in cephalopelvic disproportion that may explain the association between overweight and obesity and the increased risk of intrapartum caesarean section [19]. Obese mothers have also been reported to have slower progress and longer duration of the labour than normal weight mothers [25], which may lead to intrapartum caesarean section.

Few studies have investigated the risk of pre-pregnancy BMI with caesarean section stratified by gestational weight gain. Our study found that obese women had a higher risk of caesarean section in all categories of gestational weight gain, in line with a previous study including women in the US [17]. In that study, the risk of caesarean section was nearly three-fold in obese women with gestational weight gain below recommended range and nearly two-fold in obese women with gestational weight gain within or above recommended range compared to normal weight women. Similarly, we found that the highest risk of caesarean section was in obese women with gestational weight gain below the recommended range and overweight women had an increased risk of caesarean section only in the subgroup below the recommended weight gain range. The reasons for the findings are not clear. However, it seems that inappropriate gestational weight gain among overweight and obese women increases the risk of certain complications that may lead to caesarean section. In a previous meta-analysis, a low gestational weight gain among overweight women was associated with low birth weight and preterm birth, yet not with increased risk of caesarean section [14]. In our study, the association remained also after taking maternal and gestational age, such as gestational age, into account. In another study, higher pre-pregnancy BMI and lower gestational weight gain were associated with the risk of infant asphyxia [26], which could lead to emergency caesarean section.

We observed that gestational weight gain above the recommended range was associated with a higher risk of intrapartum caesarean section. However, this association was evident only among normal weight and overweight women. The association between a higher gestational weight gain and the risk of caesarean section among normal weight women has also been previously reported [18, 19]. A possible explanation for the finding is that excessive gestational weight gain increases the risk for cephalopelvic disproportion in normal weight women [19]. Moreover, in a previous study, we reported that among normal weight Icelandic women, the risk of pregnancy complications, including gestational hypertension, gestational diabetes, and pre-eclampsia, started to increase after gestational weight gain above 18 kg [27]. Some of these complications, in turn, have been found to increase the risk of caesarean section [28, 29]. However, in that study, we found no association between gestational weight gain with complications in delivery, including caesarean section [27]. According to that study, the Icelandic recommendation for normal weight women’s gestational weight gain has been set to 12–18 kg [30]. Therefore, Icelandic women gaining slightly above the current IOM recommendation of 11.5–16 kg may not have an intervention. However, our current study suggests that concerning the risk of caesarean section, gestational weight gain within the current IOM recommendation and Nordic Nutrition Recommendation, 11.5–16 kg [11, 12], may be optimal. A previous review showed that interventions, especially dietary interventions, aiming to manage weight gain during pregnancy have successfully reduced gestational weight gain with no maternal or fetal adverse effects [31]. Moreover, women are often willing to change their lifestyle habits into healthier ones during pregnancy [32]. Counselling a healthy diet during pregnancy could potentially decrease excessive gestational weight gain and related pregnancy and delivery complications, not only for obese women but also for normal weight and overweight women [33].

A strength of this study is its large population sample. Data were obtained from national registries, including all singleton term deliveries in Iceland from 2003 to 2014. We were able to exclude multiple and pre-term deliveries and adjust for several possible confounding factors that could have modified the observed associations. Moreover, we conducted the study in Iceland, where the rate of caesarean sections is among the lowest in the world [1]. This is mainly due to the strict policy of conducting caesarean sections only because of medical indications. This environment enables studying the associations of pre-pregnancy BMI and gestational weight gain with caesarean sections only related to medical reasons. Accurate recent measurements of actual pre-pregnancy BMI are seldom available, and self-reported pre-pregnancy BMI is prone to errors. BMI measurements before 13 weeks of gestation have thus been suggested to provide a reasonable estimate of pre-pregnancy BMI due to an average minimal weight change during these weeks [34]. Since we did not have information on actual pre-pregnancy BMI, we used the BMI measurement of the first maternal health visit occurring between gestational weeks 8 and 12. According to clinical guidelines in Iceland, a midwife or a nurse should measure maternal height and weight at the first maternal health visit [35]. A limitation of the study is that we only had data on BMI of the first and the last maternal health visits. Therefore, we could calculate only the total gestational weight gain, not the weight gain trend through the trimesters.

In conclusion, because women who are obese before pregnancy have the highest risk of caesarean section regardless of gestational weight gain, it is important to provide lifestyle interventions to prevent and treat obesity in women before conception. However, women who are normal weight or overweight before pregnancy and gain weight above the recommended range during pregnancy may also have an increased risk of caesarean section. Therefore, monitoring gestational weight gain and counselling during pregnancy should also be available for women with normal pre-pregnancy BMI to minimise the risk of caesarean section. Moreover, overweight women may also have a higher risk for pre-labour caesarean section if their gestational weight gain is lower than currently recommended.
